# Harnessing nanotechnology for cancer treatment

**DOI:** 10.3389/fbioe.2024.1514890

**Published:** 2025-01-20

**Authors:** Jiajun Zhu, HaeJu Lee, Ruotong Huang, Jianming Zhou, Jingjun Zhang, Xiaoyi Yang, Wenhan Zhou, Wangqing Jiang, Shuying Chen

**Affiliations:** ^1^ Department of Laboratory Medicine, Huashan Hospital, Fudan University, Shanghai, China; ^2^ Shanghai Medical College, Fudan University, Shanghai, China; ^3^ Department of Rehabilitation Medicine, The Sixth People’s Hospital Affiliated to Shanghai Jiao Tong University School of Medicine, Shanghai, China

**Keywords:** nanotechnology, cancer treatment, targeted drug delivery, early detection, cancer diagnostics, therapeutic strategies

## Abstract

Nanotechnology has become a groundbreaking innovation force in cancer therapy, offering innovative solutions to the limitations of conventional treatments such as chemotherapy and radiation. By manipulating materials at the nanoscale, researchers have developed nanocarriers capable of targeted drug delivery, improving therapeutic efficacy while reducing systemic toxicity. Nanoparticles like liposomes, dendrimers, and polymeric nanomaterials have shown significant promise in delivering chemotherapeutic agents directly to tumor sites, enhancing drug bioavailability and minimizing damage to healthy tissues. In addition to drug delivery, with the utilization of tools such as quantum dots and nanosensors that enables more precise identification of cancer biomarkers, nanotechnology is also playing a pivotal role in early cancer detection and diagnosis. Furthermore, nanotechnology-based therapeutic strategies, including photothermal therapy, gene therapy and immunotherapy are offering novel ways to combat cancer by selectively targeting tumor cells and enhancing the immune response. Nevertheless, despite these progressions, obstacles still persist, particularly in the clinical translation of these technologies. Issues such as nanoparticle toxicity, biocompatibility, and the complexity of regulatory approval hinder the widespread adoption of nanomedicine in oncology. This review discusses different applications of nanotechnology in cancer therapy, highlighting its potential and the hurdles to its clinical implementation. Future research needs to concentrate on addressing these obstacles to unlock the full potential of nanotechnology in providing personalized, effective, and minimally invasive cancer treatments.

## Graphical Abstract

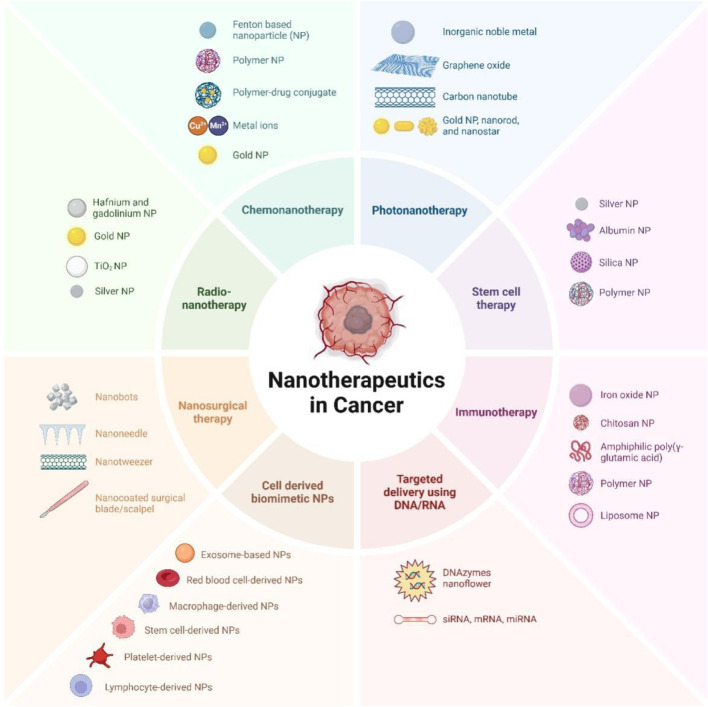

## 1 Introduction

One of the leading causes of mortality worldwide, cancer accounts for millions of deaths annually. Conventional therapies like chemotherapy, radiation therapy and surgery are often non-specific, therefore, leading to destruction of not only cancerous cells but also healthy tissues, resulting in significant side effects. Chemotherapeutic agents, for instance, lack the ability to differentiate between malignant and normal cells, thus, leads to systemic toxicity, immune suppression and poor quality of life for patients (Lau et al., 2004). Moreover, development of drug resistance in tumors presents as a major hurdle in the long-term management of cancer. These challenges underscore the urgent need for more precise, effective and less toxic therapeutic approaches to cancer.

Over the past few years, nanotechnology has become a prospective solution to the challenges posed by traditional cancer therapies (Chen S. et al., 2023). Nanotechnology, defined as the control and manipulation of materials at the atomic or molecular level, typically involving materials with dimensions of 1–100 nm, offers unprecedented opportunities to improve cancer diagnosis, treatment and monitoring. By harnessing this unique physical, chemical and biological properties of nanomaterials, researchers have been able to develop novel therapeutic and diagnostic tools that provide enhanced targeting, reduced toxicity and improved therapeutic efficacy in the clinical field (Gong et al., 2019). The application of nanotechnology in cancer treatment, commonly known as nanomedicine, involves the use of nanocarriers for drug delivery, nanoscale diagnostic tools for early detection and the design of nanomaterials for targeted therapeutic interventions. The versatility of nanomaterials enables the encapsulation and targeted delivery of various therapeutic agents, including chemotherapeutic drugs, genes, proteins and small molecules, directly to the tumor site, thereby increasing their effectiveness while reducing off-target side effects (Sajjadi et al., 2021; Ouyang et al., 2023). In addition, nanotechnology enables a concept known as “theranostics,” where these multifunctional nanoparticles combine therapeutic and diagnostic capabilities (Shailendrakumar and Tippavajhala, 2022). This combined capability enables real-time tracking of treatment responses and a more personalized approach in cancer therapy.

## 2 Nanotechnology in drug delivery for cancer treatment

### 2.1 Nanoparticles as drug carriers

**FIGURE 1:**
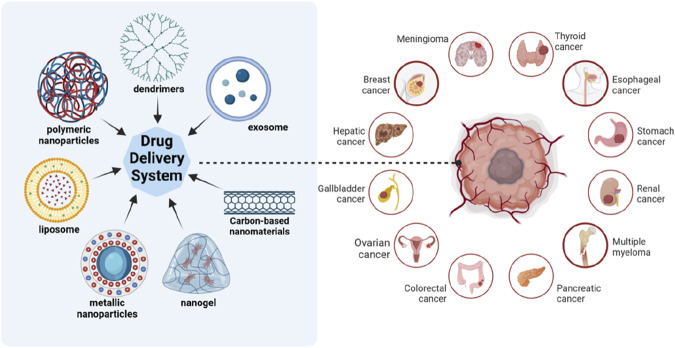
Different kinds of nanoparticles as drug carriers. Nanoparticles used for drug delivery include liposomes, polymeric nanoparticles, dendrimers, metallic nanoparticles, nanogel, carbon-based nanomaterials, exosomes and so on. They can be used to treat cancers of various organs. Created in BioRender.com.

Nanoparticles (NPs) serve as one of the most promising tools in the realm of medication delivery, particularly for cancer treatment. These nanoparticles, including liposomes, dendrimers, metallic nanoparticles (such as gold and silver nanoparticles), polymeric nanoparticles and others, offer unique capabilities that allow for the precise and targeted delivery of chemotherapeutic agents (Figure 1). Primary strength of NPs as drug carriers lies in their ability to encapsulate drugs and transport them directly to tumor cells, ensuring a higher concentration of the therapeutic agents to the targeted site (Wang and Zhang, 2023; Chen W. et al., 2023). This maximizes the efficacy of the drug and significantly reduces the systemic toxicity that is commonly associated with conventional chemotherapy (Aftab et al., 2018). One of the key reasons why nanoparticles are so effective in targeting tumor cells is their ability to take advantage of the enhanced permeability and retention (EPR) effect (Kalyane et al., 2019). Due to abnormal angiogenesis induced by cancerous cells, abnormal vasculature enables accessible penetration even to large molecules and retention of those large molecules due to incomplete immune system constructed around them. The ability to penetrate allows nanoparticles to naturally gather in the tumor microenvironment and its ability to retain within the tumor microenvironment due to poor lymphatic drainage in tumors leads to prolonged drug exposure at tumor site (Li et al., 2014). This passive targeting mechanism is particularly advantageous in solid tumors, where it is challenging to deliver sufficient drug doses when using traditional methods.

Liposomes, among the earliest form of nanoparticles utilized for drug delivery, are spherical vesicles made up of lipid bilayers capable of encapsulating both hydrophilic and hydrophobic drugs. Due to its similar structures to that of human cell membrane and encapsulation, liposome poses great advantage in its size, biocompatibility, biodegradability, low toxicity and immunogenicity (Chen et al., 2024). Clinical success of liposomal formulations such as Doxil^®^, a liposomal formulation of Doxorubicin, has shown the potential of NPs to improve the therapeutic index of chemotherapeutic agents especially in breast, Kaposi’s sarcoma and ovarian cancer (Tejada-Berges et al., 2002). Compared with free doxorubicin, liposomal formulations using polyethylene glycol-coated liposomes resulted in a decreased volume of distribution, an extended intravascular circulation half-life, and a slower plasma clearance rate. This characteristic reduces the cardiotoxicity typically associated with free doxorubicin, hence allowing patients to receive effective cancer treatments with fewer side effects. Doxil, being the first nanotechnology-based formulation approved by FDA in 1995 and one of the most mature form of nanotechnology-based drugs, is still utilized today to treat different cancers such as ovarian cancer, acute lymphoblastic leukemia, breast cancer, AIDS-related Kaposi’s sarcoma and acute myeloblastic leukemia (Ansar and Mudalige, 2020). The development of liposomal carriers for chemotherapeutics has laid the groundwork for the creation of more sophisticated nanocarriers capable of responding to specific environmental cues, such as temperature or pH, to release their drug cargo specifically at the tumor site.

Polymeric nanoparticles are another versatile category of drug carriers. These can be either be derived from the nature or made artificially. As these polymeric nanoparticles are often made of biodegradable materials, polymeric nanoparticles pose great advantage in the field of nanomedicine. Polymers of plant origin, for example is composed of polysaccharides of cellulose, starch, inulin and etc., soy proteins and polyesters from higher plants (Rizi et al., 2022). This also goes along with some synthetic polymeric nanoparticles like chitosan. Chitosan, a deacetylation form of chitin, currently function as one of the best drug carriers for cancer. Chitin, in its original form, is derived from marine organisms such as crab shells, insects, yeast, mollusks and so on (Idrees et al., 2020). In its original form, while biocompatible, bioactive and biodegradable, due to its low solubility, utilization was limited. Hence, researchers devised a novel way by deacetylating chitin to make chitosan. Thanks to its cationic characteristics, chitosan can be rapidly uptaken across the cell membrane. Par et al. utilized glycol-modified chitosan nanoparticles to promote internalization of doxorubicin in cancer cells and to hinder the activity of P-glycoprotein to counteract the drug resistance (Ashrafizadeh et al., 2023). This sustained release helps maintain an effective drug concentration within the tumor while minimizing peak drug levels that could cause systemic toxicity. Polymeric nanoparticles can also be designed to transport multiple drugs or therapeutic agents, offering the potential for combination therapy in a single nanocarrier.

Dendrimers, another type of nanoparticle, are highly branched, tree-like structures that can be precisely controlled in terms of size and surface functionality. Their unique architecture allows for high drug-loading capacity, and the presence of multiple functional groups on their surface that facilitate the attachment of targeting ligands or imaging agents (Pérez-Ferreiro et al., 2023). In the field of oncology, dendrimers were modified to be used as a nucleic acid carrier. Biocompatibility and specificity of nucleic acid therapy posed great advantage in cancer treatment (Wang et al., 2015). Hence, researchers thought of an innovative way to incorporate nucleic acid into certain cell. However, due to its large molecular weight and its hydrophilic nature, it was difficult to let the nucleic acid to penetrate inside the cell membrane. Conventional way of incorporating nucleic acid into certain cell was via viral and non-viral based vectors. However, clinical applications were hindered due to worries in immunological and oncological adverse effects (Mendes et al., 2017). Various development has been made and one of it was the utilization of PAMAM dendrimers for nucleic acid delivery in 1995. After its first put out, PAMAM dendrimers have been modified, being more compact and spherical (Banaei and Salami-Kalajahi, 2015). For example, fourth generation of PAMAM dendrimer was loaded with IFN-β to target malignant gliomas by modifying arginine (Bai et al., 2013). Another example would be with the dendrimers being functionalized with folic acid, which targets the folate receptors overexpressed on certain cancer cells, thereby improving the precision of drug delivery (van Dongen et al., 2014). Thus, dendrimers can be modified in such way to improve their biocompatibility and reduce its potential toxicity, making them an ideal candidate for delivering chemotherapeutic agents or gene therapies.

In addition to these widely studied NPs, metallic nanoparticles, including gold and silver nanoparticles, have also shown potential in cancer treatment. Among various metallic nanoparticles, gold nanoparticles gained most interest due to their biocompatibility, stability and its high electron content, enabling them to have low chemical reactivity (Kesharwani et al., 2023). The optical properties and photothermal properties of gold can be modified with various ligands to target specific cancer cells, and when irradiated with near-infrared light, generate heat, allowing them to destroy cancer cells while sparing surrounding healthy tissue. Currently, gold nanorods (GDNDs), a type of capsule-like nanoparticle is one of the thriving nanoparticles recently. This dual functionality enables gold nanoparticles to become a powerful tool in cancer therapy, offering both therapeutic and diagnostic (theranostic) capabilities. Moreover, nanoparticles can be engineered to react to specific stimuli in the tumor microenvironment, such as changes in pH (Li et al., 2016; Janic et al., 2016; Tao et al., 2024), temperature (Fu et al., 2023), or the presence of enzymes (Li N. et al., 2017). For example, pH-sensitive nanoparticles can discharge their drug payload in the acidic environment of the tumor, thereby enhancing drug release at the site of action while minimizing release in healthy tissues (Ye et al., 2018). Similarly, enzyme-sensitive nanoparticles can release their contents in response to enzymes that are overexpressed in the tumor microenvironment, further increasing the specificity and effectiveness of the treatment (Andresen et al., 2010).

### 2.2 Targeted delivery system

**FIGURE 2:**
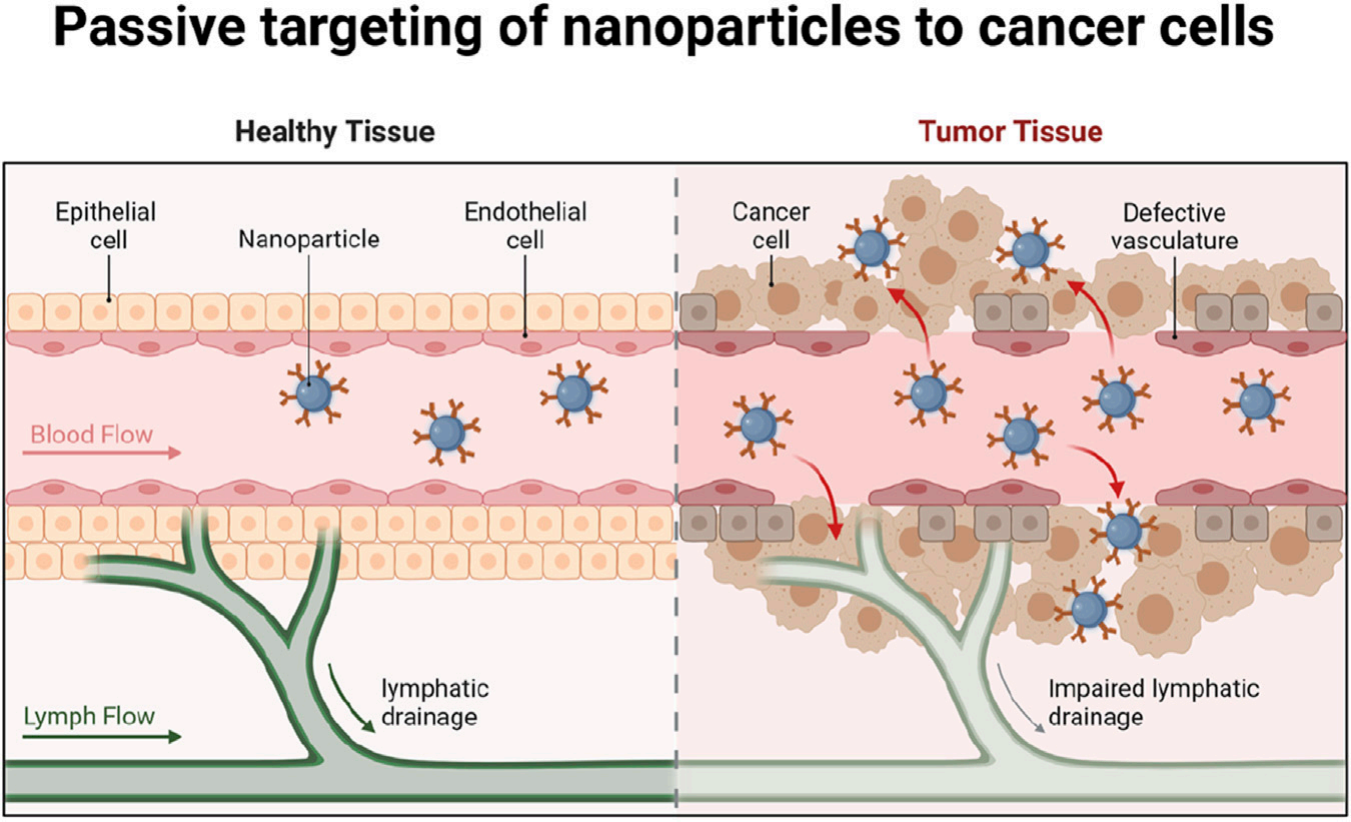
Passive targeting of nanoparticles to cancer cells. Modification of nanoparticles with ligands such as peptides, antibodies, aptamers or small molecules can precisely target and kill cancer cells and reduce the destructive effects on healthy tissues. Created in BioRender.com.

Targeted delivery systems represent a major advancement in nanotechnology-based cancer therapy, offering the potential for highly specific and effective treatment with minimized side effects (Figure 2). Functionalization of NPs with ligands like peptides, antibodies, aptamers, or small molecules enables precise targeting of cancer cell surface receptors, which are often overexpressed or uniquely expressed in tumor cells compared to normal cells (Lin and Zhang, 2023; Zhang Q. Y. et al., 2020). This selective targeting not only enhances the accumulation of therapeutic agents in cancer cells but also improves the overall efficacy of the treatment while reducing off-target toxicity to healthy issues.

**FIGURE 3:**
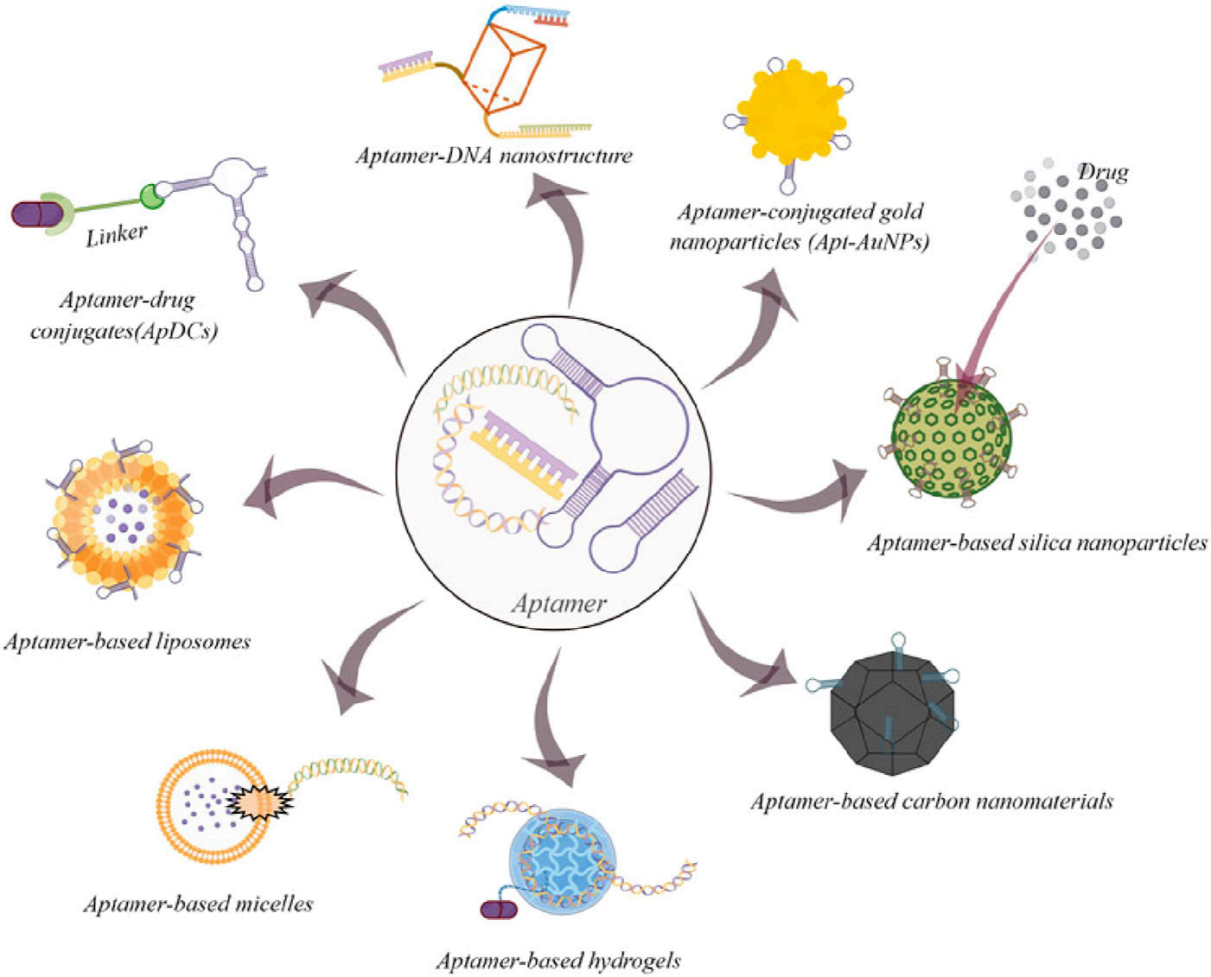
Some common examples of aptamer-based drug delivery systems for cancer therapy (By figdraw). Copyright^©^ 2022 Gao, Yin, Chen, Guo, Hu and Su (CC-BY).

A key benefit of targeted delivery systems is their capacity to exploit the molecular differences between cancer cells and normal cells. Many types of cancer cells overexpress specific receptors or proteins on their surface, which can be exploited as binding sites for ligand-functionalized nanoparticles. For example, the HER2 receptor is overexpressed in certain types of breast cancer cells, and nanoparticles functionalized with anti-HER2 antibodies can specifically bind to these receptors, facilitating the direct delivery of chemotherapeutic agents to the cancer cells (Sadat et al., 2015). This method increases drug accumulation at the tumor site while reduces the exposure of non-cancerous cells to the cytotoxic effects of the treatment, thus minimizing side effects. Another common target for ligand-functionalized nanoparticles is the folate receptor, which is overexpressed in various types of cancers, including ovarian, breast, and lung cancers (Chen et al., 2011; Boss and Ametamey, 2020). Folate-conjugated nanoparticles can attach to the folate receptor with high affinity, allowing for selective uptake of the nanoparticles by cancer cells. Once inside the tumor cells, the nanoparticles can release their therapeutic payload, resulting in enhanced drug delivery and improved therapeutic outcomes. This type of targeting is particularly advantageous because the folate receptor is either minimally expressed or absent in most normal tissues, further enhancing the specificity of the treatment (Shulpekova et al., 2021). Peptide-based ligands are also widely used for targeting nanoparticles to cancer cells. While peptides are more vulnerable to degradation and lower affinity than the molecular antibodies, peptides exhibit great advantage which are its fast and deep penetration and its efficient internalization (Vadevoo et al., 2023). Hence peptides have been devised in the field of nanotechnology to guide nanoparticles in recognizing specific receptors or proteins on the surface of cancer cells. Not only does it guide the nanoparticles, but due to their direct interactions of nanoparticles and peptides, nanoparticles are able to prevent degradation of peptides from proteases within cell. For instance, RGD peptide, which recognizes integrins overexpressed on the surface of tumor endothelial cells, has been frequently used to functionalize nanoparticles for targeted delivery to tumors. Hence, RGD peptide has been modified to be associated with nanoparticles. The modified RGD peptide known as internalized RGD (iRGD), having been modified, not only was it able to bind to integrins but it was also able to increase tissue penetration. This facilitated the penetration of the drugs into the tissue (Chen Y. J. et al., 2022). By attaching to vβ3 integrins on the endothelial cells involved in tumor cell angiogenesis, it was able to prevent the growth of new blood vessels (Chen and Chen, 2011). This approach is especially useful for targeting the tumor vasculature, as integrins are crucial in the angiogenesis process, which enables tumors to form new blood vessels and support their growth. By delivering therapeutic agents directly to the tumor vasculature, peptide-functionalized nanoparticles can inhibit angiogenesis and starve the tumor of its blood supply, effectively limiting its growth and metastasis (Crisp et al., 2014). RGD peptide was also co-administer certain drugs into cancerous tissue to activate certain endocytic pathway and induce apoptosis of the cancerous cell (Ruoslahti, 2017). In addition to antibodies and peptides, aptamers—short single-stranded DNA or RNA molecules—can also be used to functionalize nanoparticles for targeted cancer therapy (Liu et al., 2013) (Figure 3). Aptamers have a high binding affinity for specific molecular targets due to their special structure and can be selected through a process called SELEX (Systemic Evolution of Ligands by Exponential Enrichment) to recognize and bind to unique cancer cell markers (Xi et al., 2014). One advantage of using aptamers is that they are relatively easy to synthesize and modify, and they can be designed to target a wide range of cancer-related proteins. Generally, there exist two classifications of aptamers based on their structures, nucleic acid and peptide aptamers. Nucleic Acid aptamers (NA-Apts) consist of short single stranded (20-100 bps) DNA or RNA oligonucleotides that fold into 3D conformation (Mahmoudian et al., 2024). Peptide aptamers (P-Apts) were introduced after the development of nucleic acid aptamers. With the peptide aptamer embedded in an adamant protein structure, it allows high binding affinity than the free peptide, enabling it to selectively bind onto a specific target (Liu et al., 2024). Due to its safety issues, aptamers emergence into cancer therapy still possesses much challenges. Currently, AS1411, a 26-nucleotide guanine-rich DNA aptamer, entered clinical trials. It mainly functions to prevent proliferation of tumor cells by sending down certain signaling pathways (Ireson and Kelland, 2006). One advantage of using aptamers is that they are relatively easy to synthesize and modify, and they can be designed to target a wide range of cancer-related proteins.

The functionalization of nanoparticles with ligands not only facilitates targeted binding to cancer cells but also enhances cellular uptake and internalization (Kazemi et al., 2022). Once the nanoparticles bind to the target receptor on the cancer cell surface, they are typically internalized through receptor-mediated endocytosis, a process by which the cell engulfs the nanoparticle into an intracellular vesicle. This ensures that the therapeutic agent is delivered directly into the cancer cell, where it can exert its cytotoxic effects. For example, antibody-functionalized nanoparticles targeting the transferrin receptor are internalized via clathrin-mediated endocytosis, allowing for the efficient delivery of chemotherapeutic drugs to the intracellular environment of the tumor cells (Chithrani and Chan, 2007). Another critical advantage of targeted delivery systems is their ability to overcome the limitations of traditional chemotherapy, particularly drug resistance. In many cancers, drug resistance develops through mechanisms such as the overexpression of drug efflux pumps (e.g., P-glycoprotein) that expel therapeutic agents from the cancer cells before they can exert their cytotoxic effects. By functionalizing nanoparticles with ligands that specifically bind to cancer cell receptors, it is possible to bypass these resistance mechanisms. Once the nanoparticles are internalized through receptor-mediated endocytosis, the drugs can be released inside the cell, circumventing the efflux pumps and increasing drug retention in the cancer cells (Bartczak et al., 2012). Professor Mo Ran and colleagues from China Pharmaceutical University reported a nanotherapeutic strategy that employs nanoparticles co-loaded with the differentiation-inducing agent all-trans retinoic acid and the chemotherapeutic drug camptothecin to eliminate cancer stem cells (CSCs) within tumors. This approach reduces stemness-associated drug resistance, thereby enhancing chemotherapy response, inhibiting tumor growth, and preventing postoperative tumor recurrence and metastasis (Shen S. Y. et al., 2021). Furthermore, ligand-functionalized nanoparticles can be engineered to react to specific conditions in the tumor microenvironment, including pH shifts, temperature changes, or enzyme activity (Lee et al., 2017). Tumor tissues often exhibit a more acidic environment compared to normal tissues due to the high metabolic activity of cancer cells. pH-responsive polymers are widely used in chemotherapy containing nanomaterial components due to their ability to exploit the acidic microenvironment of tumors. These polymers are generally stable at physiological pH (7.4), but undergo structural changes such as swelling, dissociation, or degradation in the acidic tumor microenvironment (pH ∼6.5–6.8) or endosomal/lysosomal compartments (pH ∼4.5–6.5). This pH-triggered response can help to control the release of chemotherapeutic drugs specifically at the tumor site, improve the bioavailability of the drug, and minimize off-target toxicity (Kim et al., 2008). Polymers containing acid-labile bonds (e.g., hydrazone or imine) or functional groups such as carboxyl or amine, which are protonated under acidic conditions, thereby improving drug release efficiency. Similarly, enzyme-sensitive nanoparticles can be designed to release their cargo in response to enzymes that are overexpressed in tumors, such as matrix metalloproteinases (MMPs), which break down the extracellular matrix, promoting tumor invasion and metastasis (Li N. et al., 2017; Zong et al., 2024). By incorporating these stimuli-responsive features, targeted delivery systems can further enhance the specificity and efficacy of cancer treatment.

**FIGURE 4:**
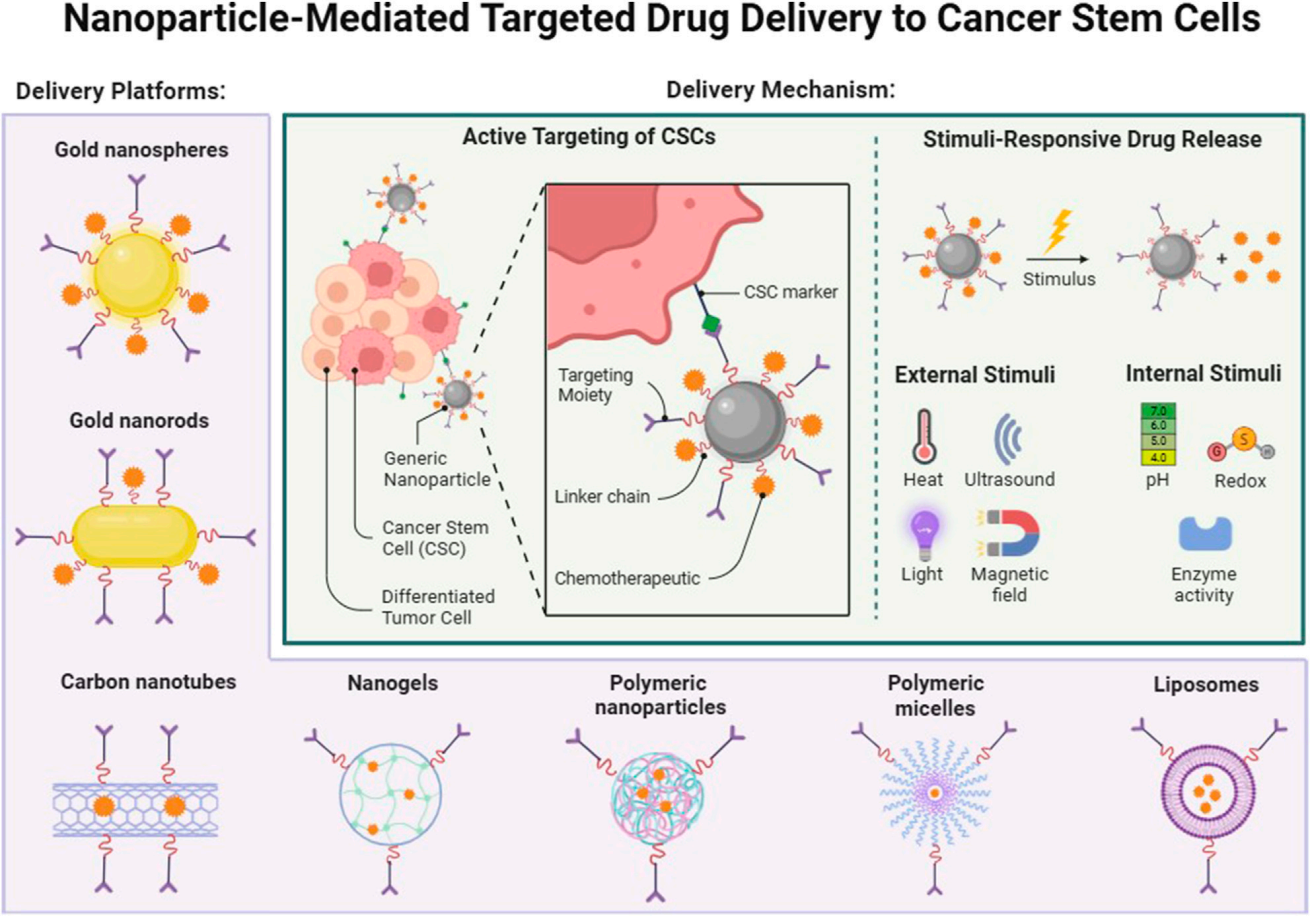
Nanoparticle-Mediated Targeted Drug Delivery to Cancer Stem Cells. Targeting tumor stem cells as an example, seven different delivery vectors are introduced, which can be targeted to activate drug release through different stimuli *in vivo* or *in vitro*. Created in BioRender.com.

Although targeted delivery systems have significant advantages, their delivery efficiency is affected by multiple factors, including surface modification of nanomedicines, affinity of targeting molecules, heterogeneity of the tumor microenvironment, and permeability of the blood-tumor barrier. Characteristics of the tumor microenvironment such as hypoxia, high acidity, and poor vascular permeability may affect the accumulation of nanoparticles and drug release. Therefore, when designing targeted nanomedicines, optimizing these factors is crucial to improving the therapeutic effect. In addition, the delivery of targeted drugs must not only consider the selectivity of the target, but also the stability of the drug carrier, the continuity of delivery, and its interaction with the tumor immune environment to further enhance the overall therapeutic effect (Figure 4).

## 3 Nanotechnology in cancer diagnostics

**FIGURE 5:**
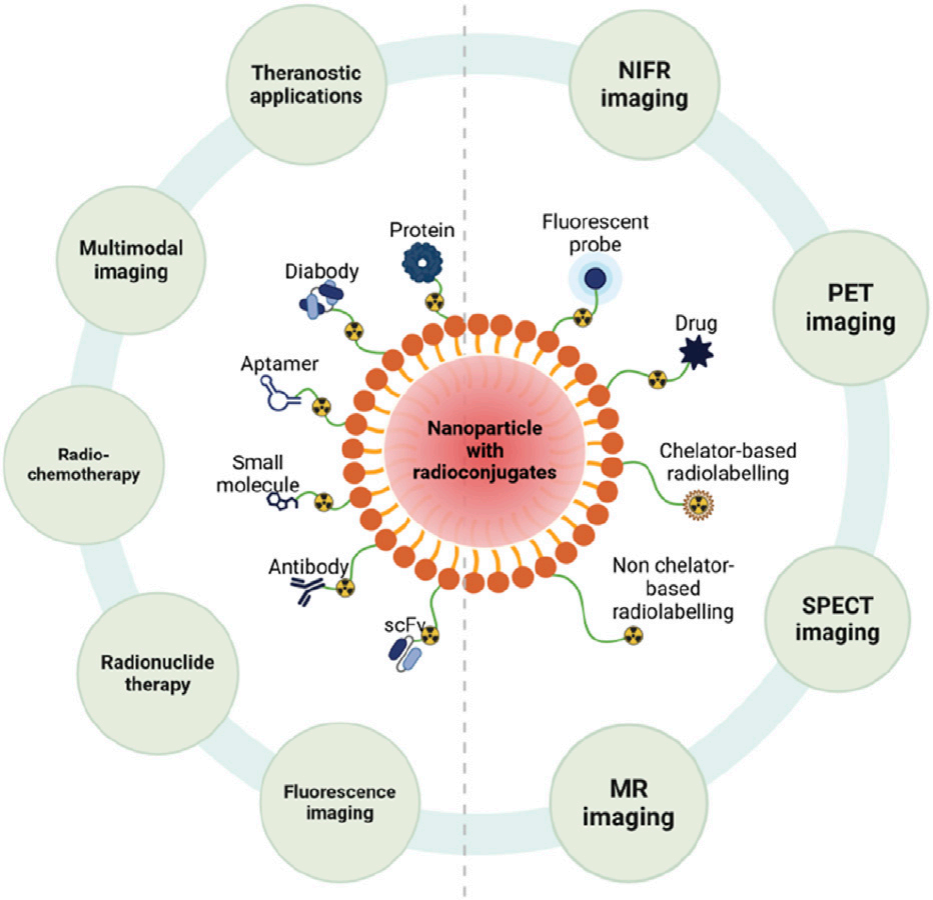
Nanoparticle-Based Radioconjugates. Ten different radioconjugates are demonstrated in this figure, each of which is used in different scenarios. Created in BioRender.com.

Nanotechnology has revolutionized cancer diagnostics by enabling earlier, more precise, and less invasive detection methods. The ability to detect cancer at an early stage is crucial for improving patient outcomes, as early diagnosis often leads to more effective treatments and higher survival rates. Traditional diagnostic techniques, such as biopsies, imaging, and blood tests, while effective, often lack the sensitivity or specificity needed to detect cancer at its earliest stages. Nanotechnology, which enables the manipulation of materials at the molecular and atomic scale, provides significant advantages in improving the sensitivity, specificity, and versatility of diagnostic tools (Khazaei et al., 2023). Nanoparticles, quantum dots, and other nanomaterials can be designed to interact with cancer biomarkers or tumor cells with high precision, leading to improved early detection and treatment monitoring (Figure 5).

### 3.1 Early detection

A key factor in effective cancer management is early detection, which can greatly enhance the likelihood of successful treatment. Nanotechnology enhances the sensitivity and specificity of early diagnostic tools, allowing for the detection of cancer biomarkers at extremely low concentrations, which is essential for identifying the disease at its earliest stages. Traditional diagnostic methods often struggle to detect low levels of biomarkers in the bloodstream or tissue samples, which can result in delayed diagnosis. Nanotechnology, however, can amplify these signals, providing more accurate and earlier detection.

Quantum dots (QDs) are considered one of the most promising nanomaterials for cancer diagnosis. Quantum dots are semiconductor nanocrystals that display distinct optical characteristics, such as exceptional brightness, photostability, and adjustable emission wavelengths (Schmidt et al., 2014). These characteristics make QDs ideal for labeling and imaging cancer biomarkers. By conjugating quantum dots with antibodies, peptides, or aptamers that bind specifically to cancer-related proteins or receptors, researchers can track the presence and concentration of these biomarkers with remarkable precision (Tsoi et al., 2013). For example, QDs can be developed to target and label proteins such as prostate-specific antigen (PSA) (Shi et al., 2008) or HER2 receptors (Chen et al., 2009; Al-Ani et al., 2021), which are overexpressed in certain types of cancer. QDs can emit fluorescence at multiple wavelengths simultaneously when excited by a single excitation wavelength, enabling multi-target and multi-channel imaging (Song et al., 2014). In cancer diagnosis, QDs of different colors can be used to label various biomolecules, allowing multi-label detection of tumors and their microenvironment. Additionally, the excitation wavelength of QDs can be tuned to the near-infrared (NIR) range (650–900 nm), which offers stronger tissue penetration, making it suitable for deep tissue imaging, such as in breast cancer and liver cancer (Zhang et al., 2008; Díaz-Garcia et al., 2024). QDs also exhibit high absorption efficiency across a broad excitation spectrum, making them compatible with common light sources like lasers or LEDs. However, in the excitation range of ultraviolet (UV) or visible light (300–500 nm), normal tissues may produce autofluorescence, reducing imaging contrast. Furthermore, using UV or short-wavelength light as the excitation source may cause tissue damage or phototoxicity (Abdellatif et al., 2022; Chen L. L. et al., 2022; Katsumiti et al., 2014). When illuminated under specific wavelengths of light, these labeled quantum dots emit bright fluorescence, making it easy to detect and visualize even minute quantities of cancer biomarkers. This allows for highly sensitive imaging, enabling clinicians to identify cancerous lesions before they become visible through traditional imaging techniques like CT or MRI (Xu et al., 2014).

In addition to QDs, nanoparticle-based biosensors offer another avenue for early cancer detection. Biosensors are analytical instruments that transform a biological response into a measurable signal, which can be electrical, optical, or thermal in nature (Luo et al., 2006). Nanoparticles, such as gold or silver nanoparticles, can be functionalized with ligands that bind to specific cancer biomarkers in blood, urine, or tissue samples. When these nanoparticles interact with the biomarker, they produce a signal that can be detected, even at very low concentrations (Liu et al., 2010). For example, gold nanoparticles conjugated with DNA or RNA aptamers can be used to detect circulating tumor DNA (ctDNA) in the bloodstream, a key indicator of early-stage cancer (Wang et al., 2016; Ding and Liu, 2024). Such biosensors offer a non-invasive or minimally invasive method for cancer screening, offering a quicker and more convenient diagnostic alternative to traditional biopsy-based techniques.

Another innovative approach is the use of nanowires and nanocantilevers for biomarker detection (Gao et al., 2023). Nanowires, due to their high surface area and sensitivity, can detect minute changes in the biochemical environment, such as the presence of specific proteins or genetic mutations associated with cancer (Li and Wang, 2013; Smith et al., 2020; Mussi et al., 2021). Nanocantilevers, which are tiny, flexible sensors, can be coated with antibodies or DNA strands that bind to cancer biomarkers. When these biomarkers are present, they cause the nanocantilever to bend, which can be detected and quantified (Vashist and Holthöfer, 2010). These nanodevices are highly sensitive and capable of detecting cancer at an early stage, offering a new frontier in point-of-care diagnostics.

### 3.2 Theranostics

Nanotechnology not only enhances the sensitivity and precision of cancer diagnostics but also opens the door to creating theranostic nanoparticles, which integrate both therapeutic and diagnostic capabilities into one platform (Chen et al., 2014; Li Y. et al., 2023). Theranostics represents a major leap forward in personalized cancer treatment, as it allows for simultaneous diagnosis, treatment, and real-time monitoring of therapeutic outcomes. This integration of therapy and diagnostics offers several advantages, including the ability to tailor treatment based on an individual’s specific tumor characteristics, monitor the reaction to therapy in real-time, and adjust the treatment plan as needed (Parveen et al., 2024; Abueva, 2021).

One of the most widely studied theranostic agents is gold nanoparticles. Gold nanoparticles (AuNPs) have unique optical and physical properties that make them ideal candidates for both imaging and therapy (Prajapati et al., 2024). Due to their strong surface plasmon resonance, gold nanoparticles can serve as contrast agents in various imaging modalities, such as computed tomography (CT), photoacoustic imaging, and optical coherence tomography (OCT) (Prajapati et al., 2024; Li C. H. et al., 2023). When modified with targeting ligands like antibodies or peptides, gold nanoparticles can specifically accumulate in tumor tissues, enhancing the contrast between healthy and cancerous tissues in imaging scans. This allows clinicians to visualize tumors with high precision (Pleckaitis et al., 2023). In addition to their imaging capabilities, gold nanoparticles can be used for photothermal therapy (PTT), a treatment that leverages the ability of nanoparticles to convert light energy into heat (Sun and Liu, 2022). When irradiated with near-infrared (NIR) light, gold nanoparticles generate localized heat, which can selectively destroy cancer cells while sparing the surrounding healthy tissue. This makes photothermal therapy a highly targeted and minimally invasive treatment option (Alba-Molina et al., 2017). The combination of imaging and therapeutic functions in a single nanoparticle system allows for real-time monitoring of the treatment’s effectiveness (Han et al., 2018). For example, after administering gold nanoparticles for photothermal therapy, clinicians can use imaging techniques to observe the extent of nanoparticle accumulation in the tumor and assess whether the heat generated is sufficient to destroy the cancer cells (Shen W. Z. et al., 2021). If necessary, the treatment can be adjusted or repeated, making the process highly adaptable to the patient’s needs.

Magnetic nanoparticles (MNPs) are another class of theranostic agents that have shown promise in cancer treatment. MNPs can function as contrast agents for magnetic resonance imaging (MRI) because of their superparamagnetic properties (Khiabani et al., 2017). At the same time, they can be used for magnetic hyperthermia, a treatment in which an alternating magnetic field is applied to the body, causing the magnetic nanoparticles to generate heat and destroy cancer cells (Molaei, 2024). This dual functionality allows for the simultaneous imaging of tumors and the application of localized hyperthermia therapy (Paez-Muñoz et al., 2023; Hedayatnasab et al., 2017). Magnetic nanoparticles can also be functionalized with drugs, enabling them to serve as drug delivery vehicles. This opens the door to combining hyperthermia with chemotherapy, providing a multi-modal approach to cancer therapy (Maksoud et al., 2022; Baig et al., 2022; Kumar and Mohammad, 2011).

Silica-based nanoparticles are yet another platform for theranostic applications. Mesoporous silica nanoparticles (MSNs) are especially appealing because of their high surface area and adjustable pore size, which allow them to carry both imaging agents and therapeutic drugs (Hao et al., 2013). These nanoparticles can be loaded with fluorescent dyes for imaging, as well as chemotherapeutic agents or gene therapy vectors for treatment (Carbonaro et al., 2016; Xu et al., 2018). Silica nanoparticles can also be modified to respond to specific stimuli in the tumor microenvironment. Alterations in pH or enzyme activity enable the regulated release of the therapeutic payload (Zhang et al., 2021; Li T. et al., 2017). The ability to simultaneously image tumors and deliver drugs in a targeted and controlled way significantly improves the therapeutic index and reduces side effects.

In addition to imaging and therapy, theranostic nanoparticles can also be used for real-time monitoring of biomarker levels in the body, providing feedback on the tumor’s response to treatment (Jirvankar et al., 2024). For instance, nanoparticles functionalized with biosensors can detect changes in the expression of specific proteins or genetic markers that are associated with drug resistance or tumor progression (Zhang J. J. et al., 2020). This real-time feedback enables clinicians to adjust the treatment regimen promptly, improving the chances of a successful outcome.

In conclusion, nanotechnology has significantly advanced cancer diagnostics by enabling early detection, precise imaging, and the development of theranostic platforms that combine diagnosis and treatment. As research in nanotechnology continues to progress, the integration of therapeutic and diagnostic capabilities into a single nanoparticle system will likely to be crucial in personalized cancer care in the future, providing more effective, targeted, and adaptable treatment options for patients.

## 4 Nanotechnology-based therapeutic strategies

Nanotechnology has revolutionized cancer treatment by introducing innovative therapeutic approaches that are more precise, effective, and targeted. These advanced methods reduce harm to healthy tissues while enhancing the treatment’s impact on tumor cells. Nanoparticles provide flexible platforms for various cancer therapies, including photothermal therapy, photodynamic therapy, gene therapy, immunotherapy, and radiotherapy enhancement. Their distinct characteristics, such as large surface area, adjustable size, and the potential for functional modifications, make them excellent tools for addressing many of the limitations found in traditional cancer treatments.

### 4.1 Photothermal therapy (PTT) and photodynamic therapy (PDT)

PTT represents a promising cancer therapy that leverages nanoparticles to turn light energy into heat to selectively target and eliminate cancer cells while sparing healthy surrounding tissues (Li et al., 2022). This highly focused treatment minimizes the side effects typically seen with conventional therapies like chemotherapy or radiation. The concept behind PTT is straightforward: nanoparticles, such as gold nanoparticles and carbon nanotubes, are constructed to absorb light—often in the NIR range—and convert it into heat (Li J. et al., 2021; Wei et al., 2021). Since cancer cells are more vulnerable to heat than normal cells, they can be selectively eliminated when the localized temperature rises beyond a critical threshold (Chen et al., 2019; Hirsch et al., 2003). AuNPs are among the most widely used materials in PTT due to their excellent optical characteristics (Sun and Liu, 2022). They exhibit strong surface plasmon resonance, allowing them to efficiently absorb NIR light and generate heat. These nanoparticles can be functionalized with antibodies or peptides, which bind to specific receptors overexpressed on cancer cells. This enables the nanoparticles to accumulate within the tumor, ensuring precise heat delivery to cancer cells when exposed to NIR light (Pornnoppadol et al., 2024). The resulting localized heating causes cancer cell destruction through mechanisms like protein denaturation and membrane disruption, while surrounding healthy tissues are largely spared due to the nanoparticles’ targeted nature (Chen and Wu, 2024). Carbon nanotubes (CNTs) are another type of nanomaterial used in photothermal therapy, valued for their unique electrical and thermal properties that make them highly efficient at absorbing NIR light and converting it into heat (Shi et al., 2022). After being introduced to the tumor site, CNTs can accumulate in the tumor via passive or active targeting, and upon NIR light exposure, they produce enough heat to kill cancer cells (Guo et al., 2023; Peng et al., 2023). Additionally, CNTs can be modified to carry drugs or other therapeutic agents, enabling a combination of photothermal therapy and drug delivery in a single treatment (Kargar et al., 2024). A major advantage of PTT is its capability to circumvent the limitations of conventional cancer treatments like radiation and chemotherapy, which often harm healthy tissues. PTT provides a more localized and less invasive treatment option, especially valuable for tumors in sensitive areas like the brain or spine. Furthermore, PTT can be repeated multiple times with minimal side effects, offering a versatile and adaptable option for treating recurring tumors.

In addition, photodynamic therapy (PDT) is a therapeutic technology that uses photosensitizers to produce reactive oxygen or free radicals under irradiation with light of a specific wavelength, thereby selectively killing diseased tissues (such as tumors or diseased blood vessels). PDT has been widely used in the treatment of cancer and certain non-cancerous diseases, offering advantages such as high selectivity, minimal invasiveness, and limited damage to normal tissues (Kim et al., 2020). Liposomal nanocarriers are widely utilized in PDT due to their ability to encapsulate hydrophobic photosensitizers. This encapsulation protects the photosensitizers from premature degradation while significantly improving their solubility in biological systems. Additionally, the small size and lipid-based nature of liposomes allow them to exploit the enhanced permeability and retention (EPR) effect, facilitating targeted accumulation of the photosensitizer within tumor tissues for greater therapeutic efficiency (Zhang et al., 2018; Feng et al., 2017). Polymeric nanoparticles are another versatile platform employed in PDT. These carriers can be engineered to achieve controlled release of the photosensitizer, reducing off-target effects and extending circulation time *in vivo*. Polymers such as polylactic-co-glycolic acid (PLGA) are commonly used due to their biocompatibility and ability to respond to environmental stimuli like pH or enzymatic activity within the tumor microenvironment (Kim et al., 2021; Wang et al., 2018; Pramual et al., 2017). Metal-based nanoparticles, particularly gold nanoparticles (AuNPs) and quantum dots (QDs), have emerged as dual-function agents in PDT. These materials not only serve as carriers for photosensitizers but also enhance light absorption, increasing ROS generation. Gold nanoparticles, for instance, exhibit strong surface plasmon resonance in the NIR region, amplifying the photochemical efficiency of the therapy. Quantum dots, with their tunable optical properties, allow simultaneous imaging and therapy, offering a theranostic advantage in cancer treatment (Calavia et al., 2018; Crous and Abrahamse, 2020; Wang et al., 2019; Li et al., 2012). Carbon-based nanomaterials, including (Lagos et al., 2022) (GO) and carbon nanotubes (CNTs), further extend the capabilities of PDT. Their high surface area allows for the conjugation of multiple photosensitizers or targeting ligands, enhancing tumor specificity. Additionally, carbon nanotubes possess unique photothermal properties, enabling the combination of PDT and photothermal therapy (PTT) in a single treatment modality. This synergy can improve therapeutic outcomes, especially in hypoxic tumors where oxygen-dependent ROS generation might be limited (Lagos et al., 2022; Zhou et al., 2014; Shi et al., 2013). The integration of oxygen-carrying nanomaterials like perfluorocarbon nanoparticles into PDT systems addresses the challenge of hypoxia within the tumor microenvironment. These carriers deliver molecular oxygen directly to the tumor site, boosting ROS production and overcoming a key limitation of conventional PDT. Similarly, mesoporous silica nanoparticles (MSNs) are gaining attention for their ability to co-deliver photosensitizers and chemotherapeutic agents, enabling combination therapy that targets tumors through multiple mechanisms (Hu et al., 2019; Gary-Bobo et al., 2012).

More importantly, PTT and PDT, as local treatment methods, have attracted widespread attention in cancer immunotherapy in recent years. These two treatment methods damage tumor cells by generating thermal effects or reactive oxygen species (ROS), thereby inducing immunogenic cell death (ICD). ICD is a cell death mode that can activate the body’s immune response, mainly through the exposure of damage-associated molecular patterns (DAMPs), such as high-mobility group protein B1 (HMGB1), ATP, and gradually exposed carnicotinic acid, triggering the immune system to recognize and eliminate tumor cells. ICD induced by PTT and PDT can enhance the antigen presentation function of dendritic cells, thereby promoting the activation of T cells and tumor-specific immune responses. In addition, when PDT and PTT are combined with immunotherapies such as immune checkpoint inhibitors and tumor vaccines, they can produce synergistic effects and further improve the effect of tumor treatment. Therefore, the combination of PTT and PDT-induced ICD mechanisms has opened up a new direction for tumor immunotherapy.

### 4.2 Gene therapy and immunotherapy

Nanotechnology has revolutionized gene therapy and immunotherapy by introducing innovative systems for delivering nucleic acids and immune modulators (Chen D. Y. et al., 2023). One of the main hurdles in gene therapy is efficiently and selectively delivering therapeutic genes or gene-editing tools, such as CRISPR/Cas9, to cancer cells (Naeem et al., 2021; Yu et al., 2021). Nanoparticles have become ideal carriers for these agents due to their capacity to shield nucleic acids from degradation, enhance cellular uptake, and specifically target tumor cells. In gene therapy, nanoparticles facilitate the delivery of small interfering RNA (siRNA) (Abashkin et al., 2023; Chang et al., 2022), messenger RNA (mRNA) (Dong et al., 2023; Cai et al., 2022), or CRISPR components (Yu et al., 2021), allowing them to either silence or repair genes driving cancer progression (Liu et al., 2023). For instance, lipid nanoparticles (LNPs) are designed to encapsulate and transport siRNA or mRNA into cancer cells. These LNPs protect the nucleic acids from enzymatic breakdown in the bloodstream and ensure their effective delivery to tumors, either through passive accumulation or active targeting mechanisms (Zong et al., 2023; Kiaie et al., 2024; Liu et al., 2023). Inside the cancer cells, siRNA can reduce the expression of oncogenes, while mRNA can prompt the cells to produce therapeutic proteins that inhibit tumor growth (Goyal et al., 2022; Wang et al., 2022). It is worth noting that LNPs usually accumulate in the liver, which limits their application in non-liver tissues, especially in targeting cancer treatment. Fortunately, in addition to the direct use of LNPs, another strategy is to combine LNPs with immune cells (such as dendritic cells, T cells, etc.) to make them a natural delivery system to deliver therapeutic molecules directly to the tumor immune microenvironment. In addition, LNPs can also be used in combination with other immunotherapy methods (such as immune checkpoint inhibitors, CAR-T cell therapy, etc.). By jointly delivering immune activators or regulators, LNPs may play a greater role in activating the immune system to fight tumors. Nanoparticles are particularly promising in delivering CRISPR/Cas9 technology, which enables precise editing of cancer-associated genes (Kim et al., 2024). By using nanoparticles to carry CRISPR components, the specificity of gene editing is improved, reducing the risk of off-target effects (Glass et al., 2018). For example, gold nanoparticles coated with Cas9 proteins and guide RNA can target and correct specific genetic mutations in cancer cells, offering a precise and potentially curative approach (Qiu et al., 2020).

In cancer immunotherapy, nanoparticles play a key role in boosting the immune system’s ability to identify and eliminate cancer cells. A central strategy in this field involves immune checkpoint inhibitors, which block proteins that normally restrain immune responses against tumors. Nanoparticles can be engineered to deliver these inhibitors directly to tumors, enhancing the body’s immune response (Sanaei et al., 2021). Additionally, nanoparticles can be loaded with tumor antigens or immune-activating molecules like cytokines to strengthen immune defenses (Li E. et al., 2023; Shukla et al., 2017).

Nanoparticles are also paving the way for cancer vaccine development. By delivering tumor antigens or mRNA coding for tumor-specific proteins to immune cells, nanoparticles can stimulate the immune system to identify and eliminate cancer cells (Park et al., 2013). Lipid nanoparticles, which have shown success in mRNA vaccine delivery for infectious diseases, are now being explored for cancer immunotherapy (Reichmuth et al., 2016; Lin et al., 2024). These mRNA-based cancer vaccines can trigger strong immune responses against tumor-specific antigens, offering long-term protection against cancer recurrence.

### 4.3 Radiotherapy enhancement

Nanoparticles can significantly enhance the effectiveness of radiotherapy by functioning as radiosensitizers, which increase the absorption of radiation by tumor cells and improve treatment outcomes. Radiotherapy, a widely used cancer treatment, employs ionizing radiation to destroy cancer cells. However, a major challenge with radiotherapy is the potential harm to nearby healthy tissues, leading to considerable side effects. By incorporating nanoparticles as radiosensitizers, it becomes possible to boost the amount of radiation absorbed by the tumor while reducing the impact on surrounding healthy tissue. AuNPs are especially useful as radiosensitizers due to their high atomic number (Z = 79), which makes them more efficient at absorbing X-rays and gamma rays compared to biological tissues (Song et al., 2023). When gold nanoparticles accumulate within a tumor, they increase the local absorption of radiation, releasing secondary electrons that induce DNA damage in the cancer cells. This elevates the likelihood of tumor cell death, allowing for reduced radiation doses and minimizing the side effects typically associated with radiotherapy (Moloudi et al., 2023; Chen Y. et al., 2023). Preclinical studies have demonstrated that using gold nanoparticles as radiosensitizers improves the therapeutic outcomes of radiotherapy in various cancer models (Chen Y. et al., 2023; Marques et al., 2022; Li J. C. et al., 2023). Other nanoparticles, such as bismuth-based and titanium dioxide (TiO_2_) nanoparticles, are also being investigated for their potential as radiosensitizers. Bismuth, like gold, has a high atomic number, which makes it an excellent candidate for enhancing radiation absorption (Shahbazi-Gahrouei et al., 2023). Titanium dioxide nanoparticles, on the other hand, can produce reactive oxygen species (ROS) when exposed to radiation, which further amplifies the cytotoxic effects on cancer cells (Morita et al., 2018). By combining these nanoparticles with targeted delivery methods, the concentration of radiosensitizers in tumors can be increased, thus enhancing the overall effectiveness of radiotherapy.

Besides enhancing radiation absorption, nanoparticles can provide real-time monitoring of radiotherapy’s effects. Theranostic nanoparticles, which have both therapeutic and diagnostic capabilities, offer imaging feedback on the distribution and effectiveness of the radiation dose. For example, iron oxide nanoparticles can act as contrast agents in MRI to track how radiosensitizers accumulate in tumors, enabling clinicians to fine-tune the radiation dose and improve treatment precision (Sankaranarayanan et al., 2022; Brero et al., 2023).

Nanotechnology has opened up a wide range of innovative treatment approaches that improve the targeting, efficacy, and safety of cancer therapies. From photothermal and gene therapies to immunotherapy and enhanced radiotherapy, nanoparticles offer a versatile platform for delivering therapeutic agents and optimizing treatment results. These nanotechnology-based strategies show great potential in overcoming the limitations of conventional cancer treatments, laying the foundation for more personalized, effective, and less invasive therapies. As research and clinical development continue, nanotechnology is set to be crucial in the future of cancer treatment, promising better patient outcomes and improved life quality.

## 5 Challenges in clinical translation

While the potential of nanotechnology in cancer treatment is vast, translating these breakthroughs from the laboratory into clinical practice remains a significant challenge. Despite the impressive preclinical results and the promise that nanotechnology holds for revolutionizing cancer treatment, the path from bench to bedside is riddled with obstacles. These challenges stem from various factors, including the complexity of nanoparticle systems, their interaction with biological environments, issues related to biodistribution and toxicity, and the regulatory frameworks that govern the approval and use of nanomedicines. For nanotechnology to be successfully integrated into routine clinical practice, it is crucial to address these challenges systematically.

### 5.1 Translating nanotechnology from bench to bedside

One of the main challenges in translating nanotechnology from the research phase to clinical application lies in the complex nature of nanoparticle systems. Nanoparticles can be engineered in a multitude of ways, with variations in size, shape, surface chemistry, and functionalization. While this versatility allows for precise design and customization, it also introduces complexity when it comes to reproducibility and scalability. Nanoparticles that work effectively in controlled laboratory environments may behave unpredictably in the human body, where they encounter a much more dynamic and variable environment (Corral et al., 2024). Factors such as protein adsorption, immune system recognition, and interaction with various tissues can all influence the behavior of nanoparticles, affecting their safety, efficacy, and biodistribution (Tarannum and Vivero-Escoto, 2022).

Another issue is the difficulty in scaling up the production of nanoparticles for clinical use. Nanoparticles that are synthesized in small quantities for research purposes may not easily translate into large-scale manufacturing. Maintaining consistent quality, particle size, drug loading capacity, and surface properties on a larger scale is essential to ensure batch-to-batch reproducibility (Colby et al., 2021). Any variation in these parameters can significantly impact the stability, therapeutic efficacy and safety of the nanoparticles, posing a major challenge for clinical translation. Addressing these technical issues requires the development of standardized and scalable manufacturing processes, which introduces an additional layer of complexity to the clinical translation of nanomedicine.

### 5.2 Biodistribution and toxicity

Ensuring the biodistribution and long-term safety of nanomaterials is one of the most critical challenges in their clinical translation. Even with surface modifications and targeting ligands, nanoparticles do not always reach the tumor site efficiently (Pirollo and Chang, 2008). Factors such as tumor heterogeneity, abnormal blood flow within the tumor microenvironment, and variations in receptor expression can lead to uneven nanoparticle distribution, with some areas of the tumor receiving higher concentrations of the drug than others (Wang et al., 2023). This uneven distribution can limit the overall therapeutic efficacy of the treatment.

Moreover, the toxicity of nanomaterials, especially the toxicity to some non-target organs (liver and bone marrow, etc.), is an important challenge. The liver is one of the main organs for the metabolism and clearance of nanomaterials. Due to its rich blood supply and high activity of phagocytes, many nanomaterials tend to accumulate in the liver, which may lead to liver inflammation, oxidative stress and liver damage. Some metal oxide nanoparticles such as titanium dioxide and zinc oxide may induce apoptosis or autophagy of hepatocytes and destroy liver tissue structure. Some polymer nanomaterials used in high doses may release toxic metabolites when degraded in the liver (Li J. B. et al., 2021; Zhu et al., 2016; Kermanizadeh et al., 2012). In addition, as an important part of the hematopoietic system, the bone marrow is highly sensitive to toxic substances. Some nanomaterials, including carbon-based nanomaterials and gold nanoparticles, may interfere with the proliferation and differentiation of bone marrow-related cells, leading to immunosuppression or anemia (Patlolla et al., 2010; Zhang et al., 2011). At present, the toxicity of non-target organs such as liver and bone marrow can be reduced by surface modification, reducing the material dose and improving the degradation characteristics of the material. In addition to optimizing nanomaterials, the toxicity assessment of nanomaterials is also extremely important (Patel et al., 2021). At present, the distribution and accumulation of nanomaterials in non-target organs can be evaluated by multimodal imaging techniques such as fluorescence imaging and magnetic resonance imaging (Vojtech et al., 2015; Santelli et al., 2020). Secondly, long-term toxicity assessment is needed, especially the potential effects on non-target organs (such as kidney, spleen, heart, etc.) under chronic exposure conditions (Mohammadpour et al., 2019). The molecular mechanisms of nanomaterial-induced toxicity also need to be further explored. Oxidative stress, activation of inflammatory pathways, and organelle damage may be key processes in the occurrence of toxicity (Das et al., 2024). Finally, it is necessary to further optimize the toxicity assessment model of nanomaterials, including the construction of 3D microenvironments *in vitro* and the use of transgenic or tissue-specific models *in vivo*, so as to better evaluate the effects of nanomaterials on specific organs (Chen Q. R. et al., 2022).

In addition to long-term toxicity, the immune response to nanoparticles is another critical consideration. Some nanoparticles may trigger immune reactions, leading to hypersensitivity, allergic responses, or immune system activation. For instance, PEGylated nanoparticles, which are often employed to enhance nanoparticle stability and prolong circulation time, have been associated with the development of anti-PEG antibodies in some patients. These antibodies can accelerate the clearance of PEGylated nanoparticles from the body and reduce their therapeutic efficacy (Shiraishi et al., 2016). Thus, understanding the immunological implications of nanomaterials is crucial for designing safe and effective cancer nanotherapies.

### 5.3 Regulatory hurdles

The regulatory approval process for nanomedicines presents its own set of challenges, primarily due to the complexity of nanoparticle systems. Unlike those well-characterized traditional small-molecule drugs, which have an exact mechanism of action, nanoparticles are multifaceted and can exhibit complex behaviors depending on their size, shape, surface charge, and composition. This complexity makes it difficult to establish standardized criteria for evaluating the safety, efficacy, and quality of nanomedicines, which complicates the regulatory approval process.

Comprehensive data on pharmacokinetics, biodistribution and toxicity of new therapeutic agents are mandated by regulatory agencies such as the U.S. Food and Drug Administration (FDA) and the European Medicines Agency (EMA) before the medicines can be approved for clinical use. For nanoparticles, these evaluations are even more rigorous due to the potential for unpredictable interactions with biological systems. Additionally, the long-term safety of nanoparticles must be thoroughly assessed, particularly because many nanomaterials may persist in the body for extended periods. This necessitates long-term preclinical and clinical studies to assess the potential risks of chronic exposure to nanomaterials, which can delay the approval process and increase the cost of development (Nijhara and Balakrishnan, 2006).

Another regulatory hurdle is the lack of standardized testing methods for nanomedicines. Current guidelines for evaluating drug safety and efficacy are often insufficient to account for the unique properties of nanoparticles (Halamoda-Kenzaoui et al., 2021). For instance, traditional assays for assessing drug release, toxicity, and pharmacokinetics may not be applicable to nanomedicines due to their complex behavior in biological environments. Consequently, the development of new testing protocols and guidelines specifically tailored to nanomedicines are required. This necessitates collaboration between regulatory agencies, researchers, and industry stakeholders to ensure that appropriate standards are established for the evaluation and approval of nanoparticle-based therapies.

The cost and time associated with regulatory approval are also major barriers to the clinical translation of nanotechnology. Nanomedicine development often requires significant investment in research, testing, and manufacturing, which can be prohibitively expensive for many small biotech companies and research institutions. Furthermore, the long timelines required for regulatory approval, particularly for first-in-class nanomedicines, can postpone the introduction of new treatments to the market. These financial and temporal constraints can hinder the development and commercialization of innovative nanoparticle-based therapies, limiting their availability to patients in need (Dordevic et al., 2022).

### 5.4 Ethical considerations and public acceptance

In addition to technical and regulatory challenges, ethical considerations and public perception also play a role in the clinical translation of nanotechnology. The use of nanoparticles in medicine raises concerns about patient safety, particularly regarding the long-term effects of exposure to nanomaterials. Patients may be hesitant to undergo treatments that involve nanotechnology due to fears about the potential unknown risks associated with these new materials. This can lead to resistance or skepticism toward nanomedicine, particularly if the risks and benefits are not communicated clearly by healthcare providers (Hall et al., 2012). To address these concerns, it is essential to engage in transparent communication with the public and ensure that patients are fully informed about the potential risks and benefits of nanoparticle-based therapies. Additionally, ethical considerations related to the environmental impact of nanomaterials must be taken into account, particularly with regard to the disposal of nanoparticles after their use in medical applications. Ensuring that nanomedicines are safe for both patients and the environment is a critical aspect of responsible nanotechnology development.

While nanotechnology holds immense potential to revolutionize cancer treatment, the clinical translation of these innovations is not without its challenges. Addressing issues related to nanoparticle stability, biocompatibility, toxicity, regulatory approval, and public acceptance is essential for ensuring that nanomedicines can be safely and effectively integrated into clinical practice. With continued research, collaboration, and the development of standardized regulatory frameworks, it is possible to overcome these hurdles and unlock the full potential of nanotechnology in cancer therapy.

## 6 Future perspectives

Nanotechnology represents the frontier of cancer treatment, offering a paradigm shift from conventional one-size-fits-all therapies to highly personalized and targeted treatments. The ability to design nanoparticles with precision and tailor them to the specific characteristics of an individual’s tumor is a key advantage that nanotechnology brings to oncology. This personalized approach has the potential to drastically improve treatment efficacy while minimizing side effects, addressing some of the most pressing challenges in cancer care today. As our understanding of tumor biology deepens and as new engineering techniques emerge, the future of nanotechnology in cancer treatment looks exceedingly promising.

One of the most exciting prospects in the future of nanotechnology is its role in personalized cancer therapy. Tumors are highly heterogeneous, not only across patients but even within the same tumor mass, with different regions exhibiting varying genetic mutations, receptor expression levels, and responses to therapy. Nanotechnology can address this heterogeneity by providing platforms for precision medicine, where nanoparticles are engineered to carry multiple therapeutic agents targeting different pathways within the tumor. For instance, a single nanoparticle could be designed to deliver a combination of chemotherapy drugs, gene therapies, and immune-modulating agents, each targeting a different aspect of tumor growth and progression. This multi-functional approach increases the likelihood of eradicating the tumor, even in cases where it has developed resistance to traditional monotherapies.

Advances in nanoparticle engineering will be crucial in realizing the full potential of nanotechnology in cancer care. The next-generation of nanoparticles will likely be even more sophisticated, incorporating a range of features such as stimulus-responsiveness, multi-drug loading, and real-time monitoring capabilities. Stimulus-responsive nanoparticles, for example, are designed to release their therapeutic cargo in response to particular environmental triggers, including the acidic pH of the tumor microenvironment, elevated temperature, or the presence of specific enzymes. These nanoparticles offer controlled drug release at the tumor site, reducing the risk of systemic toxicity and improving the therapeutic index. Moreover, future advancements in surface engineering will enable the development of nanoparticles that can more effectively evade the immune system and prolong circulation time, ensuring that a larger proportion of the drug reaches the tumor.

Another crucial area for future researches lies in the integration of nanotechnology with other emerging technologies including artificial intelligence (AI), big data, and genomics. By combining nanotechnology with AI-powered diagnostic tools, it will be possible to develop highly accurate algorithms that predict which nanoparticle formulations will be most effective for individual patients based on their tumor’s genetic profile and response to previous treatments. AI can also assist in optimizing nanoparticle design by analyzing large datasets from preclinical and clinical studies to identify the most promising nanoparticle properties for specific cancer types (Soltani et al., 2021; Adir et al., 2020; Dessai et al., 2024). The integration of genomics will further enhance this personalized approach, allowing clinicians to select nanoparticles that target specific genetic mutations or molecular pathways driving tumor growth in each patient. This convergence of technologies will enable a new era of precision oncology, where treatments are customized to the unique genetic and molecular landscape of each patient’s cancer.

Tumor biology research will continue to inform the development of more effective nanoparticle-based therapies. As our understanding of the tumor microenvironment, cancer stem cells, and metastatic processes improves, nanotechnology can be further refined to exploit these vulnerabilities. For example, targeting cancer stem cells—often the root cause of recurrence and metastasis—represents a promising application of nanotechnology. Nanoparticles can be engineered to selectively deliver therapeutic agents that eliminate these resistant cancer cell populations, potentially preventing tumor relapse after treatment. Similarly, nanoparticles can be designed to penetrate the dense extracellular matrix surrounding tumors, improving drug delivery to the tumor core, where traditional therapies often fail to reach.

Overcoming current limitations in clinical applications is crucial for the broader adoption of nanotechnology in routine cancer care. One of the key hurdles is the long-term safety and biocompatibility of nanomaterials. In the future, new biodegradable and bioresorbable materials will be developed to ensure that nanoparticles are safely metabolized and eliminated from the body, minimizing concerns about long-term toxicity or accumulation in vital organs. Moreover, researchers will continue to explore novel routes of administration for nanoparticle-based therapies, including inhalable or injectable formulations that can target and deliver drugs straight to the tumor site with minimal invasiveness.

Another future direction lies in the creation of theranostic nanoparticles, which integrate therapeutic and diagnostic capabilities. Theranostics allows for real-time monitoring of treatment efficacy, enabling clinicians to adjust therapies on the fly based on how well the tumor is responding. This level of adaptability is particularly important in aggressive or treatment-resistant cancers, where rapid adjustments to the therapeutic regimen can make a critical difference in patient outcomes. Future theranostic platforms will likely integrate a range of diagnostic modalities, such as MRI, PET, and optical imaging, with therapeutic functions like drug delivery, photothermal therapy, or immunotherapy, offering a highly personalized and responsive treatment approach.

Nanotechnology’s role in immunotherapy is also expected to grow. As cancer immunotherapy continues to gain traction, nanotechnology offers novel ways to boost the immune system’s capacity to combat cancer. In the future, nanoparticles could be utilized to deliver cancer vaccines that activate the immune system to identify and target tumors. These vaccines could be personalized for each patient, carrying tumor-specific antigens that trigger a targeted immune response. Additionally, nanoparticles can deliver immune checkpoint inhibitors or other immune-modulating agents in a more precise and controlled manner, improving the overall efficacy of immunotherapies. The potential of combining nanotechnology with immunotherapy to create long-lasting, durable anti-tumor immune responses holds significant promise for the future of cancer treatment.

Collaboration between clinicians, researchers, and regulatory bodies will also be crucial for advancing the clinical adoption of nanotechnology. The future will likely see a more collaborative approach to nanomedicine development, where regulatory agencies work closely with scientists and clinicians to develop streamlined pathways for the approval of nanoparticle-based therapies. As more nanomedicines progress through clinical trials, the accumulation of safety and efficacy data will help refine regulatory frameworks, making it easier for future nanomedicines to gain approval and reach patients more quickly.

The future of nanotechnology in cancer therapy looks promising, with immense potential to revolutionize personalized cancer care. Continued advancements in nanoparticle engineering, a deeper understanding of tumor biology, and successful integration with other emerging technologies will accelerate the adoption of nanomedicine in clinical practice. While challenges remain, such as ensuring long-term safety and navigating regulatory hurdles, the field is moving steadily toward a future where nanotechnology plays a central role in the battle against cancer, providing patients with more effective, personalized, and less invasive treatment options.

## 7 Conclusion

Nanotechnology embodies a groundbreaking approach in the realm of cancer treatment, offering innovative solutions to address many of the limitations inherent in traditional therapies. The distinctive properties of nanoparticles, including their small size, large surface area, and capacity to be functionalized with different therapeutic agents, enable targeted delivery, enhanced bioavailability, and controlled release of drugs. This precision allows for the target delivery of therapeutic agents straight to cancer cells, minimizing damage to surrounding healthy tissues and reducing systemic side effects.

Recent advancements in nanotechnology have resulted in the creation of various nanocarriers, including liposomes, dendrimers, and nanorods, each with specific advantages for drug delivery and imaging. These innovations are facilitating the creation of personalized treatment regimens that are tailored to the molecular characteristics of individual tumors, potentially improving the efficacy of therapies and patient outcomes. Moreover, ongoing research is exploring the integration of nanotechnology with other therapeutic modalities like immunotherapy and gene therapy, in order to further improve treatment effectiveness. The ability to combine multiple therapeutic approaches within a single nanoparticle platform holds promise for overcoming resistance mechanisms and achieving more comprehensive cancer control.

As the field continues to evolve, it is anticipated that nanotechnology will play an increasingly critical role in the development of more effective, targeted, and safer treatment options for cancer patients. The synergy between nanotechnology and oncology is expected to pave the way for novel therapeutic strategies that not only prolong survival but also enhance the quality of life for individuals battling cancer.
